# Moving forward with actionable therapeutic targets and opportunities in endometrial cancer: NCI clinical trials planning meeting report on identifying key genes and molecular pathways for targeted endometrial cancer trials

**DOI:** 10.18632/oncotarget.19961

**Published:** 2017-08-03

**Authors:** Helen J. MacKay, Douglas A. Levine, Victoria L. Bae-Jump, Daphne W. Bell, Jessica N. McAlpine, Alessandro Santin, Gini F. Fleming, David G. Mutch, Kenneth P. Nephew, Nicolas Wentzensen, Paul J. Goodfellow, Oliver Dorigo, Hans W. Nijman, Russell Broaddus, Elise C. Kohn

**Affiliations:** ^1^ Division of Medical Oncology & Hematology, Odette Cancer Centre, Sunnybrook Health Sciences Centre, Toronto, Ontario, Canada; ^2^ Division of Gynecologic Cancer, Department of OB/GYN, NYU Langone Laura and Isaac Perlmutter Cancer Center, New York, NY, United States; ^3^ Division of Gynecologic Oncology, Lineberger Comprehensive Cancer Center, University of North Carolina, Chapel Hill, CA, United States; ^4^ Reproductive Cancer Genetics Section, Cancer Genetics and Comparative Genomics Branch, National Human Genome Research Institute/NIH, MSC 8000, Bethesda, ML, United States; ^5^ University of British Columbia & BC Cancer Agency, Division of Gynecologic Oncology, Vancouver, British Columbia, Canada; ^6^ Department of Gynecology, Obstetrics and Reproductive Sciences, Yale School of Medicine, New Haven, CT, United States; ^7^ Section of Hematology-Oncology, Department of Medicine, The University of Chicago, Chicago, IL, United States; ^8^ Department of Obstetrics & Gynecology, Washington University School of Medicine, St. Louis, MO, United States; ^9^ Medical Sciences Program, Indiana University School of Medicine, Bloomington, IN, United States; ^10^ Division of Cancer Epidemiology and Genetics, National Cancer Institute, Bethesda, ML, United States; ^11^ James Comprehensive Cancer Center and The Department of Obstetrics and Gynecology, Ohio State University, Columbus, OH, United States; ^12^ Division Gynecologic Oncology, Department of Obstetrics and Gynecology, Stanford, CA, United States; ^13^ Department of Gynecology, University of Groningen, University Medical Center Groningen, Groningen, The Netherlands; ^14^ Department of Pathology, Unit 85, University of Texas M.D. Anderson Cancer Center, Houston, TX, United States; ^15^ Clinical Investigations Branch of The Cancer Therapy Evaluation Program, National Cancer Institute, Rockville, ML, United States

**Keywords:** endometrial cancer, molecular targets

## Abstract

The incidence and mortality rates from endometrial cancer are increasing. There have been no new drugs approved for the treatment of endometrial cancer in decades. The National Cancer Institute, Gynecologic Cancer Steering Committee identified the integration of molecular and/or histologic stratification into endometrial cancer management as a top strategic priority. Based on this, they convened a group of experts to review the molecular data in this disease. Here we report on the actionable opportunities and therapeutic directions identified for incorporation into future clinical trials.

## INTRODUCTION

Endometrial Cancer (EC) is the fourth most common cancer affecting women. Over the last decade, the incidence of EC has been increasing globally. If current trends continue, in the United States the incidence of EC will double by 2030 [1]. Furthermore, the number of women dying from EC has also been increasing disproportionally to the rise in incidence, with rates exceeding those seen for most other solid tumors [2]. There are few therapeutic options for women diagnosed with recurrent or metastatic EC, and median overall survival (OS) is short. No new agents have been approved for the treatment of EC in the past two decades [3]. New therapeutic approaches are required to meet this significant unmet need. In many other cancers, a detailed understanding of underlying tumor biology has yielded remarkable advances in therapeutic interventions, most often when agents are administered in target-selected populations. Not only does this potentially enrich for benefit for patients and clarity in interpretation, but it would also allow differentiation between presence of mutation and functionally actionable events. Applying this approach to EC is critical if we are to improve outcomes for women diagnosed with this disease.

The US National Cancer Institute (NCI) Gynecologic Cancer Steering Committee (GCSC) identified the integration of molecular and/or histologic stratification into EC management as a top strategic priority in clinical trial planning. Based on this input the NCI convened a Uterine Cancer Clinical Trials Planning Meeting (UCTPM) in January 2016. The focus of the UCTPM was to review and apply emerging molecular knowledge of EC to yield clinical trial concepts for testing actionable events in molecularly defined recurrent EC patient populations. Prior to the January meeting, a group of experts were assembled to consider the published literature focusing particularly on reports related to human specimens, with the goal of identifying evidence to support therapeutic approaches for near-term clinical trial application. Reports were generated on a number of key areas including DNA repair, hormone-related pathways, ERBB2/HER2, PI3K/AKT/mTOR signaling, the ubiquitin-ligase complex, the WNT pathway, the immune system, and obesity-driven targets. We summarize the key findings from these individual reports below and indicate reasonable candidate approaches for near-term clinical trial planning.

## MOLECULAR CLASSIFICATION OF ENDOMETRIAL CANCERS

Prognostic models for risk of metastatic disease or recurrence have been found to be of limited value in EC [4]. EC grade and histotype are major components of the models and are historically associated with poor reproducibility, with inter-observer disagreement in one third of high grade ECs [5, 6]. Thus, unlike other gynecologic cancers, it has become apparent that histologic subtype alone may not be the most effective approach to stratify and guide the treatment of EC. The traditional view of EC classification lacks the accuracy needed to sufficiently discriminate biologic variants to guide treatment.

The Cancer Genome Atlas (TCGA) project in 2013 examined nearly 500 samples of newly diagnosed endometrioid and serous ECs [7]. Detailed analysis allowed organization into four molecular subgroups based upon shared genomic features that correlated with clinical outcome. The four subgroups were: POLE-ultramutated (POLE), microsatellite instability-hypermutated (MSI), copy number low-microsatellite stable (CNL), and copy number high-serous-like (CNH). This classification is driving a paradigm shift in how EC is viewed, opening new possibilities for risk stratification, and identifying potential, actionable, subgroup-specific therapeutic targets.

**Figure 1:**
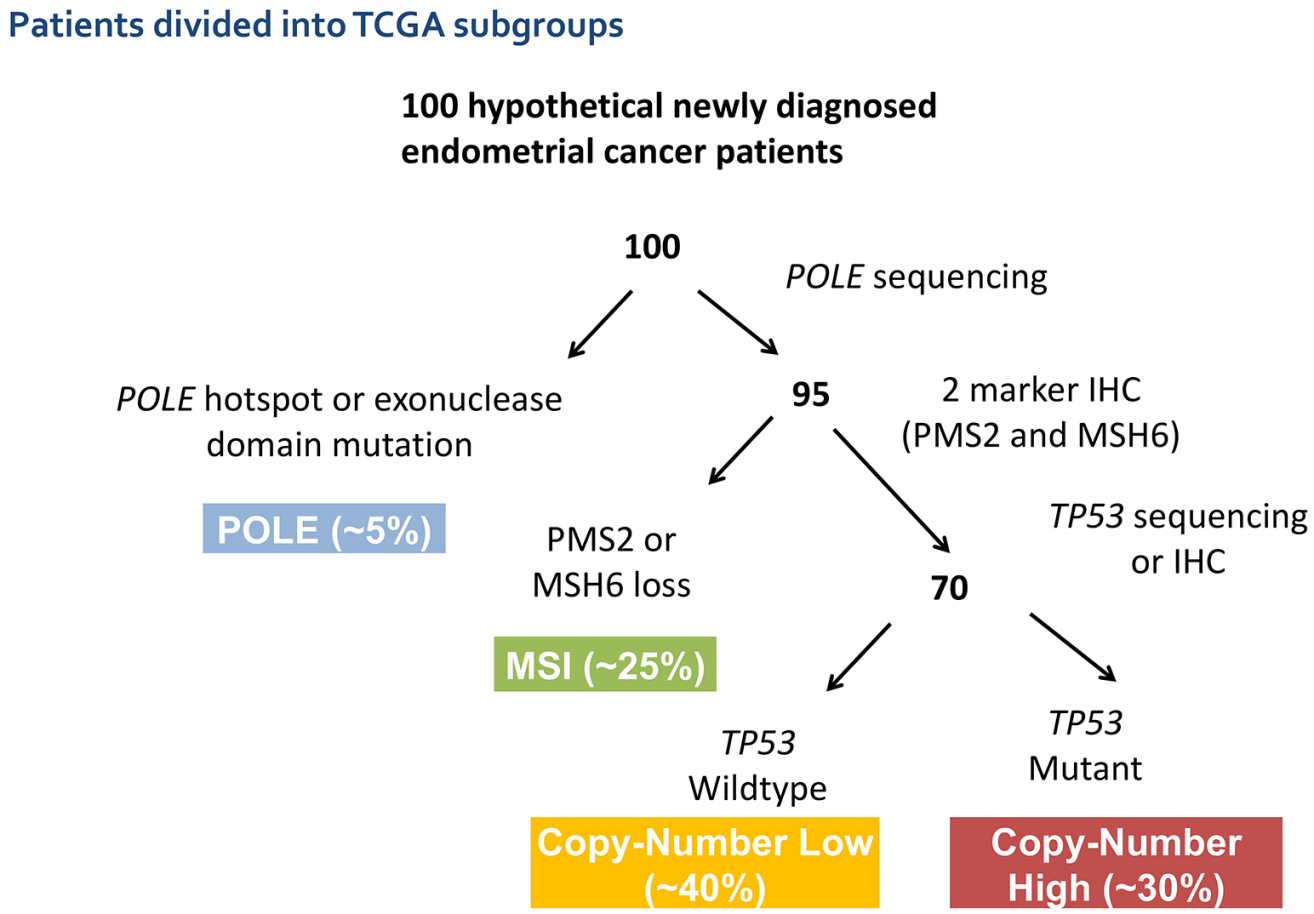
Suggested potential schema for molecular classification of endometrial cancer using sequencing and IHC results to segregate patients into the molecular subtypes previously defined by the TCGA

The POLE subgroup is characterized by a very high mutation burden driven by *POLE* exonuclease-domain mutations. This subgroup has a very favorable clinical outcome despite including tumors of varied grade and histology. The MSI subgroup is characterized by MSI and has a high mutation burden with frequent *MLH1* promoter methylation. The clinical significance of MSI in EC is uncertain, but it is frequently associated with an active immune cell infiltrate [8]. The CNL subgroup has low histological grade and frequent *CTNNB1* mutations without MSI or *TP53* mutations. *PTEN* mutations are very common in the MSI and CNL subgroups and infrequent in the CNH subgroup [7]. The CNH subgroup is defined by the presence of somatic *TP53* mutations, and included nearly all uterine serous carcinomas and ~25% of high-grade endometrioid tumors. A number of groups are developing clinically applicable classifiers to identify these molecular subgroups, which are undergoing prospective validation [9, 10] (Figure 1).

Achieving the goal to minimize therapy where it is not needed, and to tailor treatment to the cancer and patient is most likely to be achieved by incorporating molecular subgroup stratification into current classification schemas. This will allow us to prospectively test the value of such molecular subgroup stratification on treatment selection and outcome. Consideration should be given to the impact of the molecular subgroups on outcome when analyzing and interpreting existing and upcoming data from clinical trials including mixed populations of EC patients.

Serous ECs are characterized by genomic instability, high rates of somatic mutations in the *TP53, PPP2R1A, FBXW7, PIK3CA, PIK3R1, PTEN, SPOP, CHD4,* and *TAF1* genes [7, 11–16] frequent amplification and/or overexpression of the ERBB2/HER2 receptor tyrosine kinase [17, 18], and dysregulated expression of cyclin E, c-MYC, p16, E-cadherin, claudin-3, claudin-4, L1CAM and EpCAM [19]. Mutations in chromatin remodeling genes have also been reported [14, 16]. The TCGA classified 98% of serous ECs, 5% of low-grade endometrioid ECs (EECs), 19% of high grade EECs, and 75% of mixed histology ECs into a single molecular group referred to as “serous-like EC” because of their overall molecular resemblance to uterine serous carcinoma [7].

## POTENTIAL TARGETS AND THERAPEUTIC OPPORTUNITIES

### DNA repair

Classic cytotoxic agents cause DNA damage, and many newer agents lead to cell death through the inhibition of DNA repair. A major mechanism for augmentation of injury is to exploit DNA repair and cell cycle defects. Agents that prevent DNA repair or inhibit the cell cycle checkpoint cause rapid throughput in G1/S and G2/M. Such cell cycle progression results in cellular accumulation of DNA damage and subsequent apoptosis or mitotic catastrophic cell death.

The TCGA analysis identified genomic events that suggest EC, known to be susceptible to DNA damaging agents, may be affected by targeting DNA repair [7, 20]. These include: the high mutational profiles, *TP53* mutation, PTEN loss, and *ARID1A* mutations. PTEN loss of function in EC, frequent in all TCGA subgroups except CNH, may confer a homologous recombination (HR) deficiency phenotype, similar to that seen in deleterious germline *BRCA1* and *BRCA2* mutations [21]. *In vitro* sensitivity to polyADP-ribose polymerase inhibitors (PARPi) has been demonstrated in PTEN-null cell lines [21]. This remains controversial, with others finding no association with PTEN loss and response to PARPi [22]. Cell line data from colorectal and endometrial cancers suggest MSI tumors may harbor mutations in other genes involved in HR repair of double strand DNA breaks, e.g., *MRE11A* and *RAD50* [23–25]. *ARID1A* mutations are present in ~40% of MSI and CNL endometrioid tumors. ARID1A is recruited to DNA breakage sites through interaction with ATR and is required for normal G2/M checkpoint inhibition [26]. ARID1A functional loss impairs ATR activation by DNA double-strand breaks and is associated with sensitization to PARPi, and also may sensitize to platinum chemotherapy and radiation. The use of agents targeting DNA repair may also be of interest in the MSI subgroup of EC.

**Figure 2:**
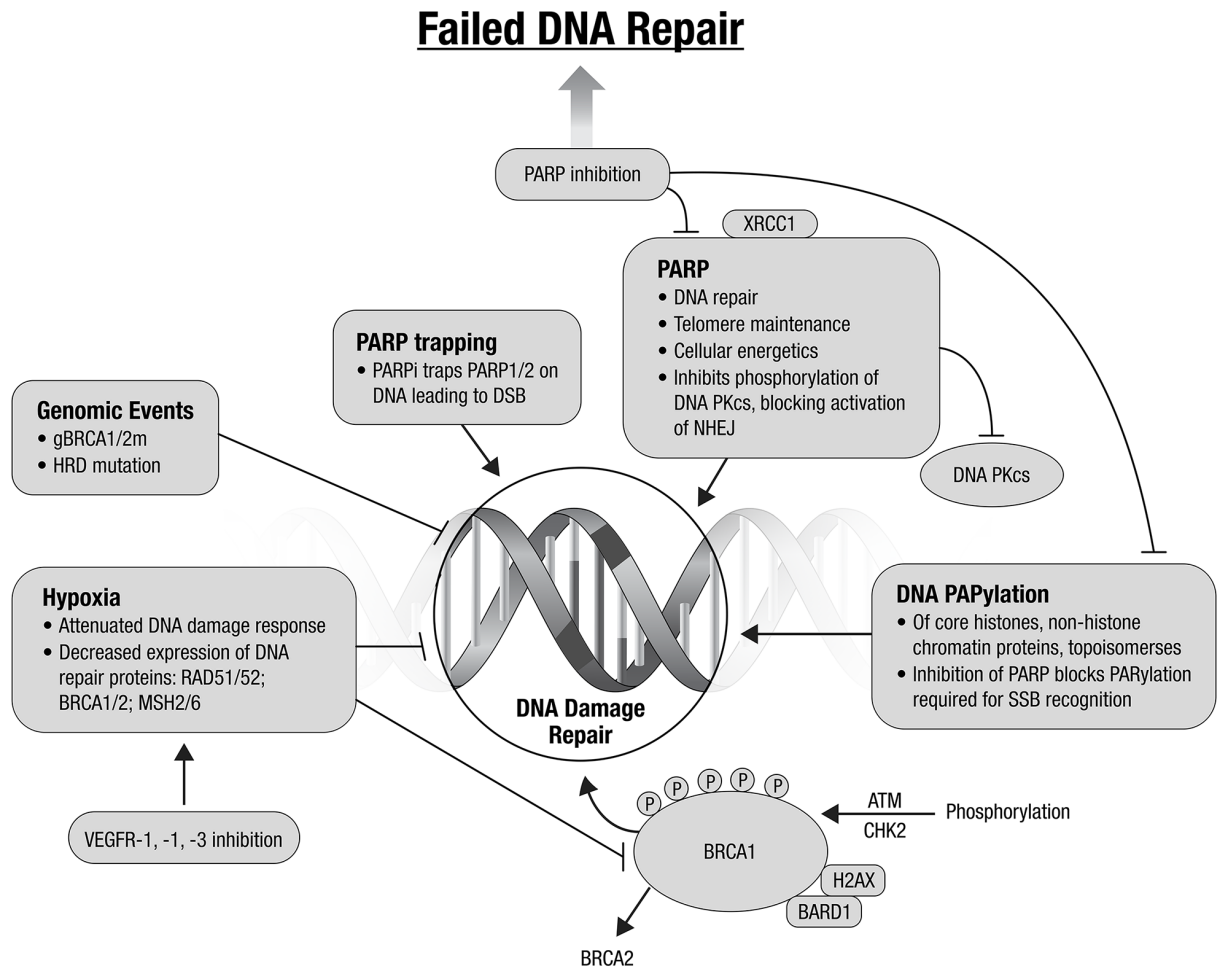
Augmenting DNA damage and repair: potential therapeutic directions (adapted from Ivy et al., 2016) [20]

The number of classes of agents targeting inhibition of DNA repair continues to expand beyond the PARPi. Promising targets include ATM and ATR, and WEE1 and CHEK1 G2 checkpoint kinases. Preclinical data suggest that combining ATR inhibitors with platinum may provide an effective treatment of platinum resistant EC [27]. WEE1 and CHEK1 are involved in the normal G2/M transition. Data to date suggest that cancers with a dependence on G2/M DNA repair may be susceptible to inhibition with DNA repair inhibitors. Combining agents targeting DNA repair is an attractive potential therapeutic strategy [20, 28–30] (Figure 2). Combinations with other targeted agents, to create context-specific synthetic lethality are also promising.

### Estrogen (ER) and progesterone (PR) receptor related pathways

Endocrine therapy has been investigated for the treatment of EC for over two decades. The 2010 Cochrane Review concluded that there was “no improvement in survival for women receiving endocrine therapy for advanced EC”. This conclusion, however, was limited by the lack of the large-scale randomized trials that would be necessary to show benefit [31]. There remains a strong rationale for endocrine therapy in EC, particularly in low grade cancers, which requires further investigation in light of our increased understanding of the EC molecular landscape.

Single agent progestins have yielded overall response rates of 20-25% [32], with some studies suggesting that ERα or PR expressing cancers are more likely to respond, although the overall data are inconsistent [32–34]. This serves to highlight the importance of standardizing tissue choice and handling, and analysis of receptor status when investigating endocrine therapy in EC. Many ECs have low levels or gradual loss of PR such that durable responses to therapy are rarely achieved. Agents targeting estrogen dependent pathways have also shown modest efficacy [35–38].

Explorations of strategies to increase hormone receptor expression are of interest. A 2004 GOG phase II study investigated tamoxifen alternating with megestrol acetate in order to increase PR expression in women with advanced and metastatic EC. A response rate of 27% was observed with a median OS of 14 months [39]. Recently *in vitro* studies suggest inducing changes in the epigenome could be a potential therapeutic strategy to increase hormone receptor expression. Differential methylation of multiple genes has been extensively reported in EC [40–43]. Aberrant DNA hypermethylation appears to be more frequent in lower grade endometrioid EC, with DNA hypomethylation a feature of serous-like EC [7]. Silencing of both *ERα* and *PR* by aberrant promoter methylation is reported in cell lines and patient samples with hypermethylation of the *ERα*-promoter C reported in over 90% and *PR*-promoter B in 70% of cases [44]. Treatment with a DNA methyltransferase inhibitor resulted in increased PR expression in several cell line studies. Histone modification also appears to influence PR expression. *PR* mRNA silencing has been shown to be reversed and functional PR expression restored with the use of epigenetic modulators in hormone-unresponsive EC cell lines [45]. HDAC inhibitors and demethylating agents are available for study and should be considered in combination with endocrine therapy.

Combining hormonal therapy with targeted therapies in rationally designed clinical trials is also an attractive therapeutic direction. Targeting the phosphoinosital-3 kinase (PI3K)/AKT/mammalian target of rapamycin (mTOR) pathway has been proposed as a mechanism for overcoming endocrine therapy resistance. Data predominantly in breast cancer suggest that cross regulation between the ER and PI3K/AKT/mTOR pathways makes targeting both pathways an attractive option [46]. Initial studies in EC with the rapalog class of mTOR inhibitors resulted in a clinical benefit rate (CBR) of 40% with an objective response rate of 32% for the combination of letrozole and everolimus [47]. The strongest indicator of lack of benefit from that treatment was serous histology. There was a statistically marginal benefit for the small subset of patients with catenin-beta 1 *CTNNB1* mutations. However, given the intra-pathway feedback loops, inter-pathway crosstalk and the incomplete blockade of the mTOR complex by the rapalogs, the limited response and duration of this combination suggests there may be further scope to optimize this strategy.

Another direction to increase hormonal therapy efficacy would be to focus on its multiple escape pathways. Provisional data from a study combining metformin, letrozole and everolimus demonstrated a CBR of 60% [48]. However, the combination of temsirolimus with alternating megestrol acetate and daily tamoxifen did not add benefit relative to hormonal therapy alone, and was associated with high rates of venous thromboembolism [49]. Exploratory biomarker work in this study and the everolimus/letrozole trial suggests that tumors harboring *CTNNB1* mutations may be more likely to respond, although responses were also seen in tumors with wild type *CTNNB1* [50].

Extrapolating from the experience in breast cancer, other options include combinations of endocrine therapy with cyclin dependent kinase inhibitors [51]. Elevated CDK4 expression has been reported in 34-77% of endometrioid ECs [52, 53]. The combination of palbociclib and letrozole is currently under investigation (NCT 02730429). Correlative studies incorporated into clinical trials will be invaluable in optimizing endocrine therapy combination strategies and patient selection.

### PI3K/AKT/mTOR pathway

The *PIK3CA-PIK3R1-PTEN* axis is somatically mutated in at least 40% of serous ECs and over 70% of endometrioid EC [7, 15, 54]. A large fraction of *PIK3CA* mutations are located in exons 1-8, in addition to exons 9 and 20 [11]. These mutations were found across all TCGA subgroups. Clinical trials of the rapalog class of mTOR inhibitors, temsirolimus, everolimus and ridaforolimus, have been completed in EC [49, 55–57]. Response rates have been modest with some patients experiencing prolonged stable disease. To date Correlative analyses of archival biospecimens have failed to identify a predictive biomarker [58, 59]. It is likely that a single or several biomarkers may be insufficient to predict clinical benefit due to complexity of this pathway and its many interactions in tumors, and the incomplete blockade of the pathway provided by these agents. There are multiple completed or ongoing single agent phase II clinical trials examining non-rapalog PI3K/mTOR agents in EC. A phase II trial (NCT01455493) of the PI3K/mTORC1/2 inhibitor, MK2206, in patients with advanced EC enrolled 56 patients, 3 patients had a confirmed clinical response and all 3 had one or more molecular abnormalities in PIK3CA, PTEN, or AKT [60]. A number of preclinical studies have shown that *HER2*-amplified serous cell lines were more sensitive to growth inhibition by mTORC1/2 inhibitors than *HER2* non-amplified serous EC cell lines, a potential future direction. Targeting the PI3K/AKT/mTOR pathway alone or in combination remains an active area of research in EC. A new study has opened to examine the role of the PI3K inhibitor, copanlisib, in patients with PI3KCA hot spot mutations in their EC. (NRG GY008/NCT02728258).

### Immune related pathways

Solid tumors frequently harbor an immune infiltrate that bi-directionally regulates cancer cell growth and metastatic potential, and in many cancers, has been demonstrated to have prognostic impact. Therapeutic immune modulation of this infiltrate could therefore be employed to optimize patient-tailored treatment and potentially outcome. The presence of tumor infiltrating lymphocytes (TiLs) and high ratios of CD8+TiLs to FoxP3+ T regulatory cells are associated in EC with a favorable prognosis [61], as are the presence of CD3+ T cells [61, 62] and CD45RO memory T cells [61]. On the other hand, the presence of CD163+ tumor-associated macrophages [63], CD4+CD25+ and CD4+FoxP3+ regulatory T cells, and high ratios of regulatory T cells to CD8+ cells were associated with a worse prognosis [64, 65]. This provides the impetus for using and understanding the role of agents that will enhance the presence of anti-tumor T-lymphocytes in the tumor tissue and microenvironment, and reduce the effect of local inhibitory factors, such as regulatory T cells.

The POLE subtype identified by TCGA has a good prognosis and displays enhanced cytotoxic T cell responses and high neoantigen load. Increased cytotoxic T cell responses are also seen in the TCGA identified MSI subgroup [8, 66]. MSI-associated EC are known to have a propensity for lower uterine segment involvement, intratumoral heterogeneity, and dense peritumoral lymphocytic infiltration [67]. Since both POLE and MSI subtypes demonstrate overexpression of PD1 and PDL1, these patients have been proposed to be excellent candidates for PD1-targeted therapies [68]. A recent meeting presentation reported 7 of 10 (70%) MSI EC responded to pembrolizumab within the KEYNOTE 158 study. There are multiple ongoing and planned trials of PD1-targeted therapy as single agents, with other immune modifying drugs, or with classic cytotoxic agents in microsatellite stable and unstable tumors. Further characterization of the immunological landscape of the copy-number low, copy-number high, and *TP53* mutant high-risk ECs may result in additional patient-tailored immunological therapies.

Antigen-specific immunotherapy aims at activating the adaptive immune system towards a specific tumor-antigen. These vaccines may be the strategy of choice for patients with low anti-tumor immune responses, as these patients do not meet the criteria for checkpoint inhibition or adoptive T cell therapy. Important criteria for target tumor-associated antigens are no or low expression in healthy tissues and overexpression in EC. Examples of tumor-associated antigens that may be targeted using this strategy are survivin and Wilms’ tumor gene 1 (WT1). In an analysis of the immunogenicity of survivin, spontaneous T cell responses were seen in 10/39 EC patients [69]. Vaccination with autologous dendritic cells electroporated with *WT1* mRNA generated a response in 3 out of 4 HLA-A2 patients, and a WT1-specific T cell response was seen in 2 of these patients [70]. It has been suggested that a combination of antigen-specific vaccines and chemotherapy may be synergistic as chemotherapy may lead to increased antigen uptake by antigen presenting cells and direct activation of dendritic cells.

### Obesity-related pathways

Obesity, diabetes and insulin resistance are associated with increased risk of developing EC and worse prognosis for incident EC [71–75]. Epidemiological evidence suggests that use of metformin, as first line treatment for type 2 diabetes, lowers cancer risk and reduces cancer deaths among diabetic patients, including women with EC [75–79]. Although the hypotheses are controversial, metformin may exert anti-tumorigenic activity through indirect effects on the metabolic milieu via cation-selective transporters and direct effects on the tumor through inhibition of mitochondrial complex 1 and subsequent AMPK activation and mTOR pathway inhibition [80, 81].

Several pre-operative window studies of metformin in EC are reported [82–85]. Each reported a statistically significant decrease in expression of Ki-67, a marker of cell proliferation, and reduction in downstream markers of the MAPK and mTOR pathways. Metformin treatment also decreased circulating plasma factors, including insulin, glucose, insulin-like growth factor-1 (IGF-1), leptin and insulin-like growth factor binding protein-7 (IGFBP7). Discrepant results have been reported on the effect of metformin on phosphorylated-AMPK [82].

There are currently many studies of metformin for endometrial hyperplasia or cancer (Table 1). These include chemoprevention in the obese patient, treatment of endometrial hyperplasia, and combination therapy with other targeted agents. An ongoing randomized, placebo-controlled phase II/III trial is designed to assess efficacy of the addition of metformin to paclitaxel and carboplatin in women with advanced and recurrent EC (GOG-0286B; NCT02065687). Secondary endpoints are to estimate differences in obesity-related parameters, cation transport, and demographics.

**Table 1:** Table Obesity pathway vs. Table Metformin studies

Center	Title	Trial type	Tumor types
UNC Lineberger Comprehensive Cancer CenterNCT01685762	Metformin for the Treatment of Endometrial Hyperplasia	Open label, safety/efficacy trial	Simple or complex hyperplasia without atypia
UNC Lineberger Comprehensive Cancer CenterNCT02035787	Metformin with the Levonorgestrel- Releasing Intrauterine Device for the Treatment of Complex Atypical Hyperplasia (CAH) and Endometrial Cancer (EC) in Non-Surgical Patients	Open label, safety/efficacy trial	Complex atypical hyperplasia and endometrial cancer
M.D. Anderson Cancer CenterNCT01697566	An Endometrial Cancer Chemoprevention Study of Metformin Versus No Treatment in Women with a Body Mass Index (BMI) >/= 35 kg/m2 and Hyperinsulinemia	Randomized, double blind, phase III efficacy trial	Endometrial Cancer
M.D. Anderson Cancer CenterNCT01797523	A Phase II, Single-Arm Study of RAD001 (Everolimus), Letrozole, and Metformin in Patients with Advanced or Recurrent Endometrial Carcinoma	Open label, phase II, safety/efficacy trial	Endometrial Cancer
Gynecologic Oncology GroupNCT02065687	A Randomized Phase II/III Study of Paclitaxel/Carboplatin/Metformin (NSC#91485) Versus Paclitaxel/Carboplatin/Placebo as Initial Therapy for Measurable Stage III or IVA, Stage IVB, or Recurrent Endometrial Cancer	Randomized, double blinded, placebo controlled, phase II/III trial	Endometrial Cancer
Queensland Centre for Gynaecological CancerNCT01686126	Improving the Treatment for Women with Early Stage Cancer of the Uterus	Randomized, open label, efficacy, phase II, Mirena IUD *versus*, Mirena IUD + Metformin *versus* Mirena IUD + Weight Loss Intervention	Endometrial hyperplasia with atypia, Endometrial Cancer
Fudan University, ChinaNCT01968317	Megestrol Acetate Plus Metformin to Megestrol Acetate in Patients with Endometrial Atypical Hyperplasia or Early Stage Endometrial Adenocarcinoma	Randomized, open label, efficacy, phase II	Endometrial hyperplasia with atypia, Endometrial Cancer

### ERBB2/ HER2

ERBB2/HER2 is a receptor tyrosine kinase that mediates signaling via the PI3K and mitogen activated protein kinase (MAPK) pathways. It is amplified in 21%-47% of serous ECs, found in the TCGA CNH subgroup, and in 3%-21% of endometrioid ECs [86, 87]. Trastuzumab is a humanized monoclonal antibody that targets HER2. Although clinical responses to trastuzumab in HER2+ serous and endometrioid EC cancer patients have been noted in case reports [88, 89], a phase II trial (GOG181B) evaluating the activity of trastuzumab for HER2+ recurrent or advanced-stage EC observed no objective responses [89, 90]. Studies in tumor tissues and serum from EC patients raise the possibility that ECs may be intrinsically resistant to trastuzumab because they have relatively high levels of the p95 variant of HER2, which lacks the extracellular domain targeted by trastuzumab [91, 92], or because of the frequent somatic activation of the downstream PI3K pathway [93]. It has therefore been hypothesized that small molecule inhibitors that bind the intracellular domain of both HER2 and p95, such as lapatinib, might be more effective than trastuzumab in EC. A phase II trial of lapatinib in unselected patients with recurrent or persistent EC observed limited clinical activity [94]. Potential explanations for the limited activity included recruitment of unselected patients, and/or intrinsic lapatinib-resistance due to activation of the PI3K pathway. Afatinib, a pan-ERBB inhibitor, is currently under investigation in a phase II clinical trial in patients with persistent or recurrent HER2+ uterine serous carcinoma (NCT02491099).

TCGA has shown that amplification of HER2 and somatic mutations of *PIK3CA*, the p110 kinase subunit of PI3K, often co-occur in serous/serous-like EC [7]. Whether these events occur within the same or distinct subpopulations of a given tumor remains to be determined and may have clinical implications, as noted for breast cancer [95]. It would seem prudent that future clinical trial design of HER2 targeted therapies in EC should include a comprehensive molecular assessment of the PI3K pathway, including PIK3CA (all exons), PTEN, and PIK3R1, the gene encoding the p85α regulatory subunit of PI3K. Combination therapy of pan-ERBB inhibitors with PI3K inhibitors has been shown to be synergistic in preclinical models of serous cancer [96]. Importantly, dual inhibition initiated after tumor progression with single agent treatment was still effective in inducing tumor regression in tumor bearing mice. Thus, dual HER2/PIK3CA blockade may represent a novel therapeutic option for EC patients harboring tumors with HER2 gene amplification and mutated *PIK3CA*.

#### WNT pathway

**Figure 3:**
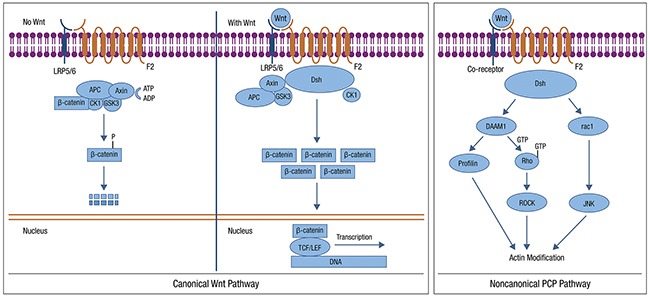
WNT Pathway: Most WNT signaling in EC occurs via the CTNNB1-dependent pathway Possible targets include: use of WNT antagonists, reduced WNT ligand secretion, increased degradation of WNT and inhibition of CDK4/6 Canonical (left) and non-canonical (right) WNT signaling are shown. This file is licensed under the Creative Commons Attribution-Share Alike 3.0 Unported license. https://en.wikipedia.org/wiki/Wnt_signaling_pathway

WNT signaling functions predominantly through both CTNNB1-dependent, and independent pathways, often termed canonical and non-canonical WNT signaling, respectively [97–99]. The WNT pathway is typically activated by one of the WNT family members binding to the frizzled (FZD) receptor to activate the disheveled (DVL) protein (Figure 3). This pathway is deregulated in many human tumors. Under normal conditions, CTNNB1 is phosphorylated by WNT pathway members and targeted for proteasomal degradation through ubiquitination, leading to active repression of CTNNB1 target genes. When the pathway is disrupted, such as somatic mutations in pathway members or *CTNNB1*, CTNNB1 is not targeted for degradation, avoids phosphorylation, and accumulates in the cytoplasm, ultimately entering the nucleus and leading to CTNNB1-mediated transcription. Secreted WNT antagonists include members of the Dickkopf (Dkk) family that prevent Wnt signaling through the low-density lipoprotein receptor-related protein (LRP). Dkk3 has been shown to be downregulated in EC and correlated with advanced stage and high-risk clinicopathologic factors. Forced Dkk3 expression *in vitro* reduced proliferation, anchorage-independent growth, and invasion [100].

WNT signaling in EC predominantly involves the CTNNB1-dependent, or canonical, signaling pathway. *CTNNB1* mutations occur in 52% of the CNL EC subgroup identified in the TCGA [7]. Alterations are uncommon among serous tumors and are present at low frequency in MSI+ tumors. Several studies have suggested that *CTNNB1* mutations are associated with a worse prognosis in early-stage low grade EC [101]. The accumulation of nuclear CTNNB1 has been demonstrated to be more common in high-grade EC and associated with loss of CDH1, suggesting more aggressive behavior [102]. There are few WNT pathway targeted agents available currently [103]; such agents would be of interest to explore in EC.

### Ubiquitin-ligase complexes

The proper regulation of cellular protein levels by the ubiquitin proteosome system is an important facet of cell biology. Dysregulation of ubiquitin mediated proteosomal degradation of cellular proteins is often observed in human cancers [104, 105]. Recent whole exome sequencing studies in EC have uncovered frequent somatic alterations in the *FBXW7* and *SPOP* genes, which encode ubiquitin ligase adaptor proteins and are more commonly seen in the CNH TCGA subgroup. This pathway is thus a novel direction for consideration. The SKP1-CUL1-FBXW7 (SCF^FBXW7^) complex is an E3 ubiquitin ligase complex that regulates the degradation of a large number of protein substrates, many of which are transcriptional regulators [106]. Several proteins that are regulated by the SCF^FBXW7^ complex, such as cyclin E, cMYC, mTOR, and MCL1 promote oncogenesis in solid tumors. *FBXW7* is a haplo-insufficient tumor suppressor [107], and is somatically mutated and/or deleted across a wide range of human cancers, with EC and T cell acute lymphocytic leukemias most frequently mutated [7, 14, 106, 108, 109]. Comprehensive sequencing studies in EC have shown that *FBXW7* mutations are more abundant in serous (15%-29%) [7, 14, 16, 109] and serous-like (21%) [7] cancers, than in either clear cell (7%-13%) [14, 110] or endometrioid (10%-27%) ECs [7, 14]. The presence of *FBXW7* mutations in concurrent cases of serous EC and serous endometrial intraepithelial carcinoma suggests these are early genetic events for this subtype [109]. In addition, *FBXW7* is also mutated in 20% of undifferentiated uterine carcinomas and in 23% of uterine carcinosarcomas [111–113].

The SPOP-CUL3-RBX1 ubiquitin ligase complex also regulates protein turnover via ubiquitin-mediated proteasomal degradation. Thus far, the repertoire of proteins that are regulated by the SPOP-CUL3-RBX1 complex appears to be largely distinct from those that are regulated by the SCF^FBXW7^ complex. Although somatic mutations in *SPOP* are rare in most human cancers, they occur at higher rates in EC [7, 14, 16], and prostate cancer [114]. *SPOP* mutations have been documented in 7%-8% of serous ECs [7, 14, 16], 5% of serous-like ECs [7], 0-9% of endometrioid ECs [7, 16], and 8% of clear cell ECs [14]. The majority of *SPOP* mutations in EC and in prostate cancer localize to the MATH domain, which binds proteins that are targeted for ubiquitination and proteasomal degradation. The MATH domain of SPOP functions in an analogous manner to the WD repeats of FBXW7, leading to the speculation that missense mutations in the MATH domain are likely to be dominant-negative or loss-of-function mutants that disrupt the binding of SPOP to one or more of its protein substrates. Functional studies of the *SPOP* mutations that have been found in EC are at a very early stage, but thus far indicate that a subset of *SPOP* mutants have an impaired ability to regulate ERα [115]. Recent work by Barberi et al., 2015 indicates that *SPOP* mutations in prostate cancer lead to defects in HR and confer sensitivity to PARP inhibition [114]. Whether this phenomenon also holds true for EC, remains to be elucidated.

### Chromatin-remodeling

Mutated chromatin-remodeling genes have been reported in primary serous EC. Whole-exome sequencing analysis of a small number of primary serous endometrial tumors (n=13), followed by targeted gene sequencing in a larger cohort of serous ECs, identified frequent somatic mutations in *CHD4* (17%), which encodes a subunit of the NuRD-chromatin-remodeling complex [14, 16]. A confirmatory study also noted frequent deletion of a small segment of chromosome 19 containing *MBD3*, another subunit of the NuRD-chromatin-modification complex, and frequent mutations in *TAF1.* The TAF1 protein has histone acetyltransferase activity and is an element of the TFIID basal transcription factor complex. Le Gallo et al., have reported TAF1 mutations in nearly 10% of clear cell EC [116]. Lower frequency mutations in several other chromatin remodeling genes including *EP300* (8%), and *ARID1A* (6%) have been noted in serous ECs [14]. *ARID1a*, a component of the SWI/SNF chromatin remodeling complex, is also frequently mutated in endometrioid ECs including all TCGA subgroups except CNH, up to 40% by different databases [7, 117]. Overactivity/overexpression of EZH2, a histone methyl transferase, downregulates the suppressor ARID1a, creating a loss of suppressor function and resulting in increased proliferation, migration, and invasion of target malignant cells. Therapeutic inhibition of EZH2 is now in early evaluation and could be targeted to *ARID1a* mutant EC [118]. The expansion of the histone deacetylase inhibitor group and development of demethylating agents may be of use in this subset of patients.

## THE PATH FORWARD FOR IDENTIFYING FUTURE ACTIONABLE OPPORTUNITIES

Clearly the emerging molecular data provide us with multiple potential avenues to pursue in terms of early phase clinical trial design for EC. In addition, TCGA identified molecular subgroups provide a potential framework for developing new risk stratification models. Actionable opportunities are those for which abnormalities have been identified and demonstrated to be biologically necessary and/or sufficient. We are now beyond the point of mutations being actionable by definition alone and require more than simple biologic plausibility. Refinement of the molecular subgroup model by combining conventional parameters, and potentially other biomarkers will add value and enhance potential for patient selection clinical trial designs. Incorporation of high quality, validated, correlative studies into our clinical trials with appropriate collection of tumor and surrogate samples will be essential to capitalize on the wealth of information that can be gained from positive and negative studies. Collaborative data sharing and access to samples for biomarker identification, and perhaps more importantly, validation will speed discovery. Lastly, translating opportunity and hypothesis into the clinic always requires consideration of other unique issues that may be found in patient populations. Endometrial cancer is now affecting younger women, though still remains a cancer predominantly of older women, many of whom have other medical comorbidities. Such clinical variables may affect the complexity of some of the combination treatment ideas that could be evaluated in EC. The clinical opportunities identified in this review illustrate the complexity of EC, and also provide us with a framework for leveraging combination strategies targeting the many interrelated pathways implicated in EC biology.

Daphne W Bell receives royalties as a co-inventor on U.S. Patent No.7,294,468 “Method to Determine Responsiveness of Cancer to Epidermal Growth Factor Receptor Targeting Treatments”, which is licensed to Esoterix Genetic Laboratories LLC.
